# Case Report: Neurosurgery, octreotide, cabergoline, radiotherapy, pasireotide and pegvisomant – a multimodal strategy for adolescent acrogigantism

**DOI:** 10.3389/fendo.2026.1954698

**Published:** 2026-10-20

**Authors:** Lukasz Dzialach, Agnieszka Bogusz-Wójcik, Elzbieta Moszczyńska, Agnieszka Wojciechowska-Luzniak, Grzegorz Zieliński, Maria Maksymowicz, Przemysław Witek

**Affiliations:** 1 Department of Internal Medicine, Endocrinology and Diabetes, Medical University of Warsaw, Warsaw, Poland; 2 Department of Endocrinology and Diabetology, The Children’s Memorial Health Institute, Warsaw, Poland; 3 Department of Neurosurgery, Military Institute of Medicine, Warsaw, Poland; 4 Department of Department of Cancer Pathomorphology, Maria Sklodowska-Curie National Research Institute of Oncology, Warsaw, Poland

**Keywords:** acrogigantism, acromegaly, pasireotide, pegvisomant, pituitary neuroendocrine tumors (PitNETs)

## Abstract

Growth hormone (GH)-secreting pituitary neuroendocrine tumors (PitNETs) are rare in the pediatric population. They often present as large, invasive tumors and are frequently associated with an underlying genetic predisposition, making their management particularly challenging. Optimal treatment requires an individualized multidisciplinary approach, which often involves a combination of surgery and adjuvant medical therapy or radiotherapy when necessary. We present a challenging case of an adolescent female with acrogigantism. The patient was diagnosed at the age of 13 years and 10 months during the evaluation of chronic headaches. Brain magnetic resonance imaging revealed an invasive PitNET measuring 38 × 23 × 32 mm, while biochemical assessment confirmed marked GH hypersecretion. The disease followed an aggressive course, and biochemical control could not be achieved despite optimal multimodal therapy. Sequential treatment included neurosurgical resection, octreotide LAR, cabergoline, pasireotide LAR, proton beam radiotherapy, and pegvisomant. Combination therapy with pegvisomant and octreotide LAR ultimately normalized insulin-like growth factor 1 concentrations while maintaining long-term radiological tumor stability. This case underscores the complexity of managing aggressive GH-secreting PitNETs in adolescents and emphasizes the need for individualized, multidisciplinary care and long-term follow-up. Combined medical therapy with pegvisomant and octreotide LAR may provide effective biochemical control when surgery, radiotherapy, and standard medical treatment are insufficient.

## Introduction

Acromegaly is a rare, chronic systemic disorder caused by autonomous hypersecretion of growth hormone (GH) and, consequently, elevated levels of insulin-like growth factor 1 (IGF-1) (1, 2). In the vast majority of cases, the disease is caused by GH-secreting pituitary neuroendocrine tumors (PitNETs) (1, 3), whereas rare cases result from ectopic secretion of GH-releasing hormone (GHRH) by non-pituitary NETs (4, 5). In the pediatric population, GH excess is referred to as pituitary gigantism or acrogigantism, with excessive linear growth and marked tall stature representing the characteristic phenotypic manifestations of the disorder (6, 7).

Acromegaly predominantly affects middle-aged individuals, with the majority of cases diagnosed during the fourth and fifth decades of life (8). Pediatric-onset cases account for less than 5% of all patients with acromegaly; however, the precise epidemiological data on GH-secreting PitNETs in children and adolescents remain scarce (9, 10).

Transsphenoidal resection of a PitNET remains the treatment of choice and offers the potential for complete cure (1). However, complete tumor resection cannot be achieved in all patients, particularly in the presence of invasive macroadenomas. Consequently, a significant proportion of patients require adjuvant treatment, including pharmacotherapy and, less commonly, radiotherapy (11). Current medical therapies target different components of the GH–IGF-1 axis and include first- and second-generation somatostatin receptor ligands (SRLs) (12–16), dopamine agonists (DAs) (17), and GH receptor antagonists (18, 19). In addition to achieving biochemical remission, surgical debulking may alleviate mass effects and enhance responsiveness to subsequent medical therapy (20, 21).

The management of GH-secreting PitNETs in children and adolescents remains particularly challenging owing to the rarity of the disease, the high prevalence of large and invasive tumors at diagnosis, and the frequent presence of an underlying genetic predisposition (1, 6, 7, 10). Therefore, treatment requires an individualized multidisciplinary approach and long-term follow-up to optimize both short- and long-term clinical outcomes (7, 10).

In this paper we present a challenging case of a female patient with adolescent-onset acrogigantism whose hormonal hypersecretion and disease-related complications required a multidisciplinary and multimodal treatment approach to achieve effective disease control.

## Case report

A 13-year-10-month-old girl was referred to the Department of Pediatric Neurology in February 2023 for evaluation of progressively worsening headaches, excessive daytime sleepiness, a 10-kg weight gain over the preceding six months, and primary amenorrhea. Headaches had first appeared in June 2022, occurring twice weekly and predominantly affecting the occipital and temporal regions. Since September 2022, they had become daily, accompanied by retro-orbital pain, and showed only a partial response to analgesics. The patient denied vomiting, loss of consciousness, visual impairment, or visual field defects. Between September and December 2022, her height increased from 175 cm to 180 cm, while her shoe size increased by two sizes over the preceding year.

Routine laboratory investigations demonstrated hyperuricemia (uric acid 7.7 mg/dL; reference range [RR]: <6.0), impaired fasting glucose (103 mg/dL; RR: 70–99), marked hyperinsulinemia (109.1 μIU/mL; RR: 3.0–25.0), and hypertriglyceridemia (242 mg/dL; RR: <115).

The head magnetic resonance imaging (MRI) performed on 20 February 2023 revealed a 38 × 23 × 32 mm sellar and suprasellar mass with intermediate signal intensity on T1- and T2-weighted images and heterogenous, avid contrast enhancement. The lesion had an irregular shape and extended into the interpeduncular and prepontine cisterns, involving the anterior pituitary, pituitary stalk, part of the hypothalamus, and the cavernous segments of the internal carotid arteries. The optic chiasm and intracranial optic nerves were displaced superiorly by the mass (**Figure 1**). No other focal intracranial abnormalities were identified.

**Figure 1:**
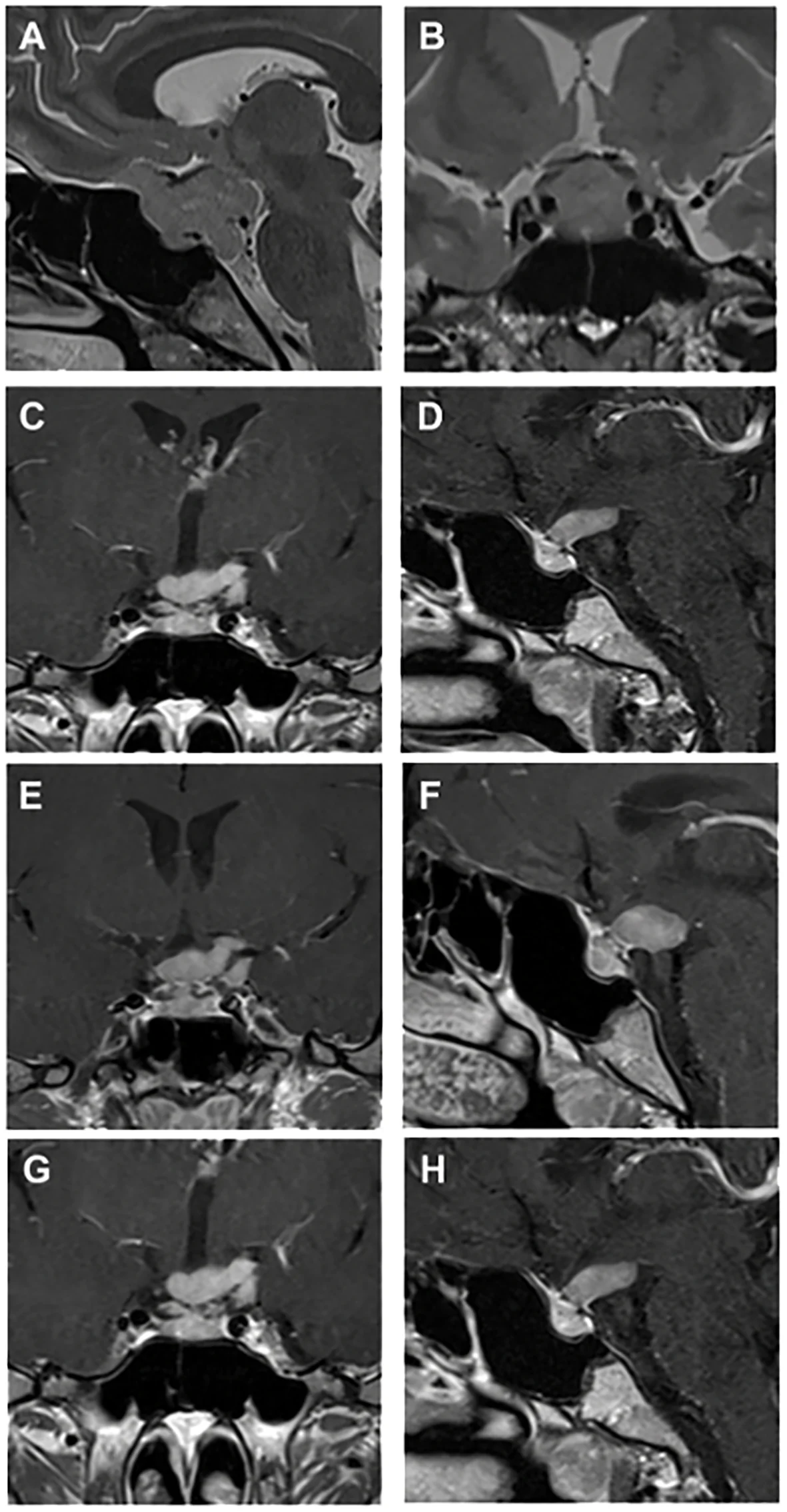
Sequential magnetic resonance imaging over the clinical course of the patient. Contrast-enhanced T2- **(A, B)** and T1-weighted **(C–H)** sagittal **(A, C, E, G)** and coronal **(B, D, F, H)** images. The timing of the scans were as follows: at clinical presentation (February 2023; **(A, B)**; after the partial resection of the pituitary tumor (November 2023; **(C, D)**; after six months of pegvisomant therapy (October 2024; **(E, F)**; following the addition of a first-generation somatostatin receptor ligand to pegvisomant therapy and radiotherapy of the residual tumor (October 2025; **(G, H)**.

The patient was subsequently referred to the Department of Pediatric Endocrinology, The Children’s Memorial Health Institute, Warsaw, Poland. The patient had been previously healthy. She was born from the first twin pregnancy at 38 weeks of gestation and had no history of systemic diseases, surgeries, or allergies. The family history was negative for malignancy. On physical examination, her height was 179.7 cm (>97th percentile; +3.03 SD), body weight was 74.6 kg (95th percentile for height), and BMI was 23 kg/m² (87th percentile). Body proportions were normal for her height. Acanthosis nigricans was present in the axillary and inguinal regions. Pubertal development had begun in 2022, but menarche had not yet occurred. Tanner stage was P4/5 and B2/3. No galactorrhea was observed. **Figure 2** shows the growth chart of the presented patient.

**Figure 2:**
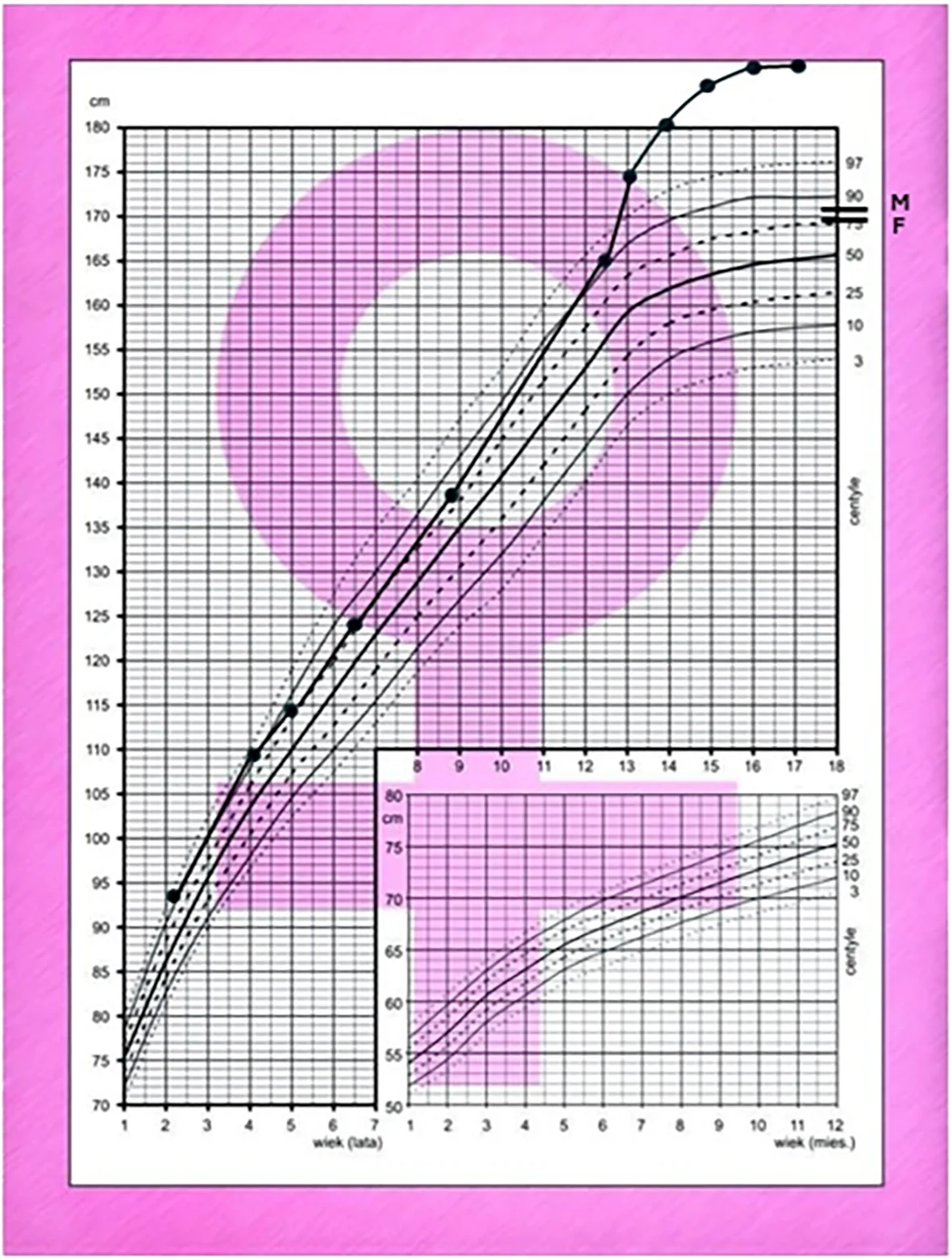
The growth chart of the presented patient.

The family history revealed tall stature on both the maternal and paternal sides. The patient’s twin sister was 172.5 cm tall (+1.84 SD; 97th percentile), weighed 65 kg, and experienced menarche in January 2023. The mother was 171.8 cm tall (+1.04 SD; 90th percentile) and had menarche at 14–15 years of age. The maternal uncle was 180 cm tall (+0.20 SD; 55th percentile). The father was 185.5 cm tall (+1.11 SD; 80th percentile). The paternal grandmother measured 173 cm (+1.25 SD; 93rd percentile), and the paternal grandfather was 180 cm tall (+0.20 SD; 55th percentile).

Hormonal evaluation revealed markedly elevated GH and IGF-1 concentrations of 86.75 ng/mL (RR: <3.33) and 977.7 ng/mL (RR: 115.9–533.4), respectively. The GH nadir during oral glucose suppression test was 78.69 ng/mL. Laboratory findings were consistent with hypogonadotropic hypogonadism, with LH <0.07 IU/L (RR: 2.2–8.1), FSH 0.66 IU/L (RR: 2.2–10.1), and estradiol 15.2 pg/mL (RR: 21–258). Hypoprolactinemia was also present, with a PRL concentration of 0.70 ng/mL (RR: 2.8–29.2). The thyrotropic (TSH: 3.289 µIU/mL; RR: 0.48–4.17, fT4: 1.26 ng/dL; RR: 0.83–1.43, fT3: 3.59 pg/mL; RR: 3.0–4.7) and corticotropic (ACTH: 49.62 pg/mL; RR: 7.2–63.3, morning serum cortisol: 22.8 µg/dL; RR: 5.27–22.45) axes were preserved. The patient underwent an ophthalmological consultation, which revealed normal visual acuity and fundus examination findings. Visual field testing demonstrated non-specific defects and fixation errors. Blood pressure monitoring and electrocardiograph were within normal limits.

Following the neurosurgical consultation, the patient was qualified for PitNET resection with preoperative preparation using a SRL. Octreotide LAR (30 mg every 28 days) was initiated. The treatment was well tolerated, with no reported adverse effects. Headaches persisted but became less severe. However, progressive enlargement of the hands and feet continued. Follow-up evaluation in May 2023, after three doses of octreotide LAR, demonstrated persistently elevated GH (107.7 ng/mL) and IGF-1 (918.5 ng/mL) concentrations. Therefore, the dose of octreotide LAR was increased to 40 mg, and cabergoline was added at a dose of 3.5 mg per week. However, subsequent follow-up evaluations over the following months showed no improvement in disease control (**Figure 3**).

**Figure 3:**
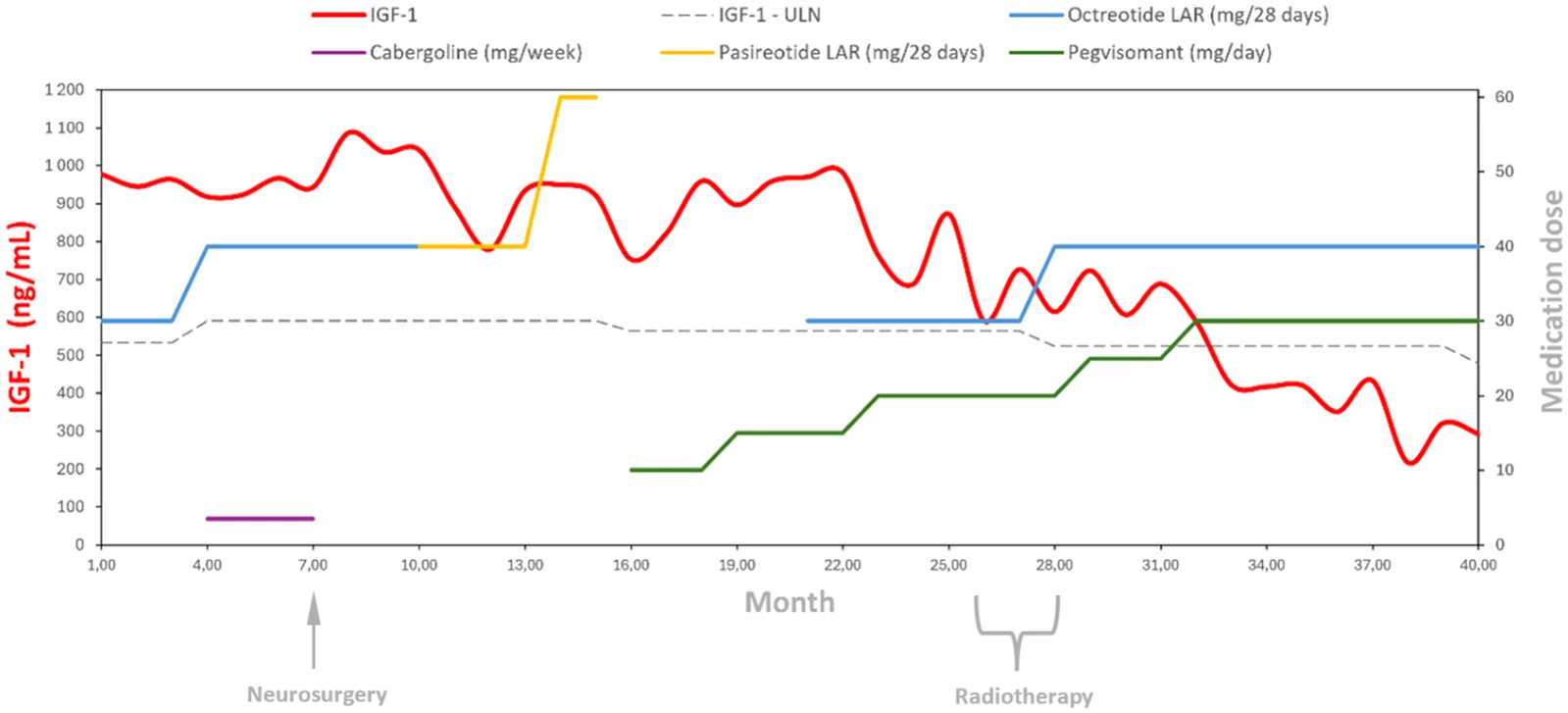
Longitudinal changes in serum IGF-1 levels from diagnosis in February 2023 to June 2026. The figure depicts the course of IGF-1 concentrations during follow-up alongside the pharmacological therapies administered, partial pituitary tumor resection, and radiotherapy.

In August 2023, the patient underwent an uncomplicated partial resection of the tumor via right frontal craniotomy. Histopathological examination confirmed a somatotroph PitNET with a Ki-67 proliferation index > 5%. Electron microscopy demonstrated ultrastructural features consistent with a sparsely granulated tumor subtype (**Figure 4**). The tumor could be classified as grade 2B (Trouillas classification).

**Figure 4:**
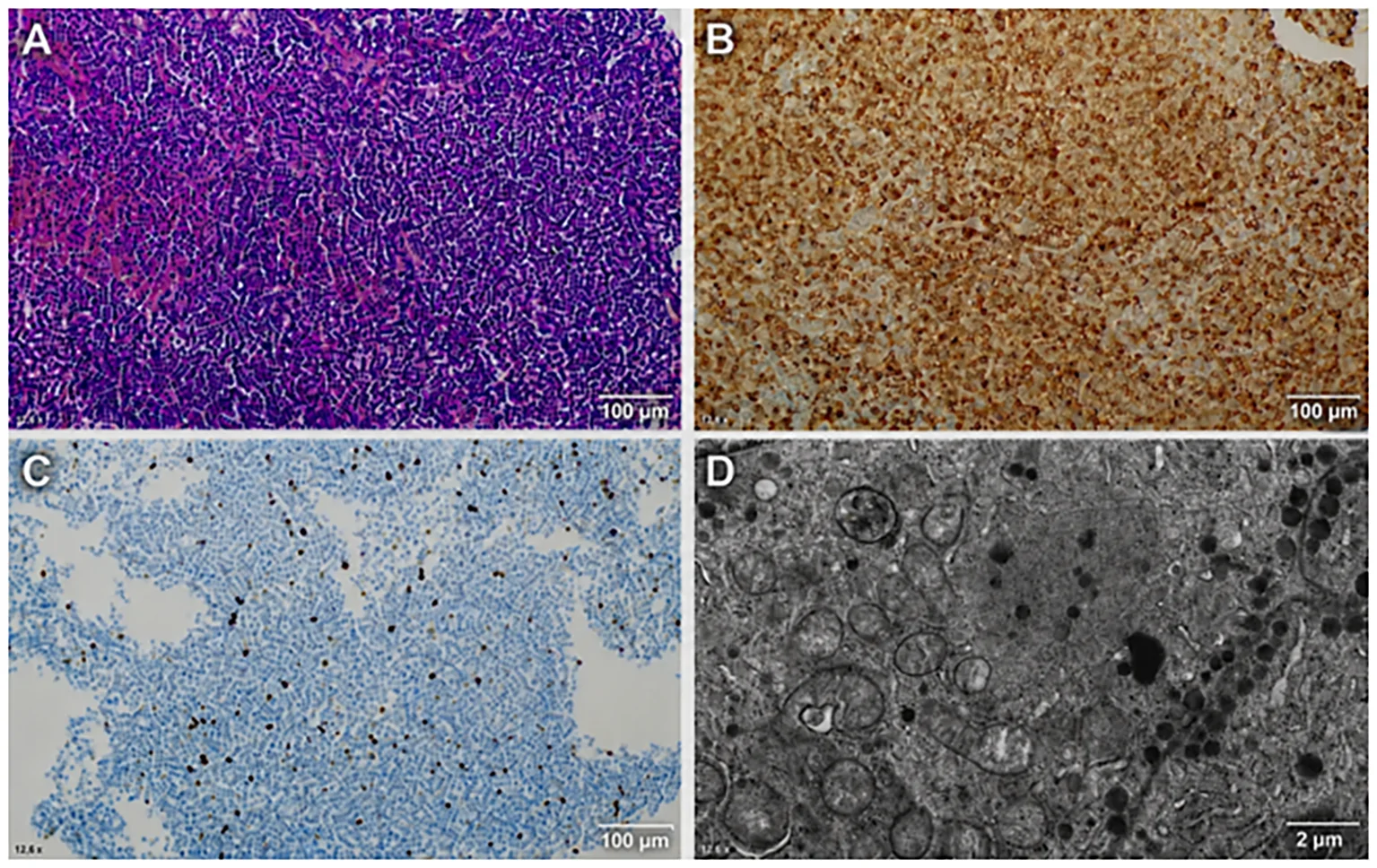
Pituitary neuroendocrine tumor (PitNET): sparsely granulated somatotroph pituitary adenoma. **(A)** Histopathological image of the PitNET (H&E staining, ×200). **(B)** Positive immunohistochemical staining for growth hormone (×200). **(C)** Elevated Ki-67 labeling index (×200). **(D)** Transmission electron micrograph (×17,500) showing a fibrous body and neuroendocrine secretory granules.

Postoperatively, persistently elevated IGF-1 (1086.5 ng/mL; reference range: 123.4–552.8) and GH (19.2 ng/mL) concentrations were observed, although GH levels had decreased markedly. Therefore, treatment with octreotide LAR (40 mg every 28 days) was reinitiated (**Figure 3**). Additionally, due to confirmed thyrotropic and gonadotropic axis deficiencies, the patient was started on levothyroxine replacement therapy and estradiol therapy. Follow-up pituitary MRI performed 3 months after surgery (November 2023) showed a marked reduction in tumor volume (**Figure 1**).

The patient underwent subsequent re-evaluation by the neurosurgical team, which concluded that radical treatment was not feasible. For further management, the patient was referred to the Department of Internal Medicine, Endocrinology and Diabetes of the Medical University of Warsaw.

In December 2023, following an inadequate response to first-generation SRL therapy, treatment with pasireotide LAR was initiated at a dose of 40 mg every 28 days and escalated to 60 mg after 3 months. However, pasireotide LAR therapy proved ineffective in achieving disease control and was discontinued after 5 months of treatment (**Figure 3**).

In May 2024, pegvisomant therapy was initiated at a starting dose of 10 mg/day and gradually escalated over the following months. Despite these dose adjustments, the initial response to treatment remained unsatisfactory. After 6 months of therapy (October 2024) follow-up pituitary MRI demonstrated slight enlargement of the residual tumor mass and a greater degree of compression/remodeling of the left optic tract compared with the previous examination (**Figure 1**). Consequently, octreotide LAR at a dose of 30 mg every 28 days was added to the treatment regimen (**Figure 3**). During the first months of combined therapy, stabilization of the patient’s linear growth was achieved.

Additionally, between February and April 2025, the patient underwent proton beam radiotherapy. The clinical target volume, encompassing the residual tumor and both cavernous sinuses, received a total dose of 50.4 Gy (RBE) delivered in 1.8 Gy (RBE) fractions, followed by a boost to the residual tumor to a cumulative dose of 54 Gy (RBE), also delivered in 1.8 Gy (RBE) fractions.

The first normalization of IGF-1 concentration was achieved after 18 months of pegvisomant therapy, in October 2025. At that time, the patient was receiving pegvisomant at a dose of 30 mg/day in combination with octreotide LAR 40 mg every 28 days (**Figure 3**).

Follow-up pituitary MRI performed in October 2025 demonstrated a slight reduction in the residual tumor mass (**Figure 1**). The latest pituitary MRI in May 2026 showed a stable tumor remnant.

During further follow-up, the function of the remaining pituitary axes did not recover. The patient continued to require levothyroxine replacement therapy (currently 150 μg daily) and sex hormone replacement therapy (currently oral estradiol 2 mg daily plus micronized progesterone 200 mg daily). Furthermore, in April 2026, approximately one year after completion of proton beam radiotherapy, the patient developed secondary adrenal insufficiency, requiring initiation of hydrocortisone and prasterone replacement therapy.

Genetic testing using next-generation sequencing (NGS) was performed in both the patient and her twin sister and revealed no germline pathogenic variants in the following genes: *AIP, APC, ATRX, CDC73, CDKN1B, DICER1, MAX, MEN1, NF1, PRKAR1A, PTEN, RET, SDHAF2, SDHB, SDHD, TMEM127*, and *VHL*.

## Discussion

We report a highly challenging case of adolescent-onset GH-secreting PitNET that required a comprehensive multidisciplinary and multimodal therapeutic strategy. Optimal disease control was achieved only after several lines of treatment, including neurosurgery, radiotherapy, and medical therapy with octreotide LAR, cabergoline, pasireotide LAR, and pegvisomant. Despite these interventions, normalization of IGF-1 levels was ultimately achieved only with combination therapy consisting of pegvisomant and octreotide LAR.

The primary goal of therapy for GH excess is the normalization of age-adjusted IGF-1 levels, which contributes to improved long-term health outcomes by normalizing growth velocity, preventing excessive height, controlling symptoms, reducing disease-related morbidity, and improving (1, 10, 11). Tumor shrinkage, as well as preservation of visual and pituitary function, are additional therapeutic goals (1, 10). A multimodal treatment approach is often required to achieve these outcomes. Children and adolescents with GH excess represent a particularly challenging patient population, as they are more likely to present with aggressive disease characterized by high GH concentrations and large tumor size at diagnosis, resistance to medical therapy, and a substantial symptom burden (7, 10, 22).

For patients with GH-secreting PitNETs, surgical resection remains the preferred first-line treatment owing to its potential to achieve complete disease remission (1, 2, 10). Remission following surgery is reported in approximately 40–60% of cases involving macroadenomas (23, 24). In the present patient, following neurosurgical evaluation and qualification for surgery, preoperative medical therapy was initiated, consisting initially of octreotide LAR and subsequently intensified with the addition of cabergoline. Current evidence does not support the routine use of preoperative medical therapy to improve postoperative remission rates (1, 25); however, selected patients with invasive adenomas may benefit from preoperative pharmacological treatment (26, 27). In the pediatric population, preoperative medical therapy may also be considered to reduce growth velocity and limit further height gain (7, 10). Despite preoperative medical therapy, no significant biochemical, radiological, or clinical improvement was observed. Serum IGF-1 concentrations remained elevated, follow-up pituitary MRI demonstrated no reduction in tumor size, and accelerated linear growth persisted. Therefore, approximately six months after diagnosis, a partial resection of the pituitary tumor was performed via a right frontal craniotomy. In acromegaly, the transsphenoidal approach is most commonly employed; however, giant tumors with substantial extrasellar growth may require a transcranial procedure (1, 28). Although surgical remission was not considered achievable in this patient at the outset, tumor debulking was performed with the aim of reducing GH hypersecretion and enhancing the response to adjuvant medical treatment. Postoperatively, octreotide LAR therapy (40 mg every 28 days) was reintroduced, but failed to achieve satisfactory disease control, thereby necessitating further lines of treatment.

Pasireotide LAR is typically used as a second-line medical therapy in adult patients with acromegaly inadequately controlled despite treatment with the maximum tolerated dose of first-generation SRLs (1). The high efficacy of pasireotide in adult patients has been demonstrated in both clinical trials and real-world studies (16, 29–32). Data regarding the use of pasireotide LAR in children and adolescents remain limited. However, it has been used in adult patients with childhood-onset GH excess, with mixed results (33–35). In the present patient, pasireotide LAR was ineffective, as neither normalization of IGF-1 concentrations nor control of linear growth was achieved. Nevertheless, follow-up MRI demonstrated stable residual pituitary tumor tissue. Pasireotide LAR was well tolerated and did not result in any clinically significant deterioration of glucose metabolism. This observation is consistent with previous reports indicating that younger patients tend to exhibit more favorable glycemic responses to pasireotide LAR therapy (36).

Due to the lack of response to SRLs therapy, pegvisomant was subsequently initiated at a dose of 10 mg/day and uptitrated at approximately 3-month intervals. Pegvisomant is a recombinant GH analogue that blocks GH receptors and reduces IGF-1 production (19, 37–39). Although experience remains limited, several studies and case reports on the use of pegvisomant in pediatric patients have been published (9, 40–44). However, in the presented patient the initial response to treatment was unsatisfactory, with persistently elevated IGF-1 concentrations. Follow-up pituitary MRI performed after approximately 6 months of treatment revealed a slight increase in the size of the residual tumor mass, accompanied by more pronounced compression of the left optic tract. Although tumor enlargement during pegvisomant therapy likely reflects the natural history of the adenoma rather than reduced IGF-1–mediated feedback, careful radiological monitoring is warranted, given the lack of direct pituitary action of pegvisomant (11, 45). In this case it was therefore decided to reintroduce octreotide LAR in combination with pegvisomant. Following a multidisciplinary team discussion, the patient was also qualified for proton beam fractionated radiotherapy targeting the residual tumor mass. Long-term follow-up data remain limited and are insufficient to conclusively demonstrate the superiority of one radiotherapy modality over another (6, 46, 47). The selection of radiotherapy modality should be guided by patient age and preferences, tumor size and characteristics, proximity to critical structures, and the availability, efficacy, and tolerability of alternative medical therapies (48). Nevertheless, fractionated external beam proton therapy has been widely adopted in the pediatric population because of its lower incidence of late toxicities. Proton beam therapy further reduces radiation exposure to tissues outside the target volume, and is therefore expected to decrease the risk of long-term adverse effects (6, 10, 49, 50). Data on the use of Gamma Knife radiosurgery in pediatric patients with acromegaly are limited. In a study involving 12 pediatric patients with acromegaly, biochemical remission was achieved in 42% of patients during the follow-up period (46).

The first time that normalization of IGF-1 levels was achieved in October 2025 during combined therapy with pegvisomant at a dose of 30 mg/day and octreotide LAR at a dose of 40 mg every 28 days. Combination therapy with pegvisomant and SRLs has also been successfully reported in pediatric patients with GH excess (43, 44, 51). This therapeutic approach is particularly recommended for patients requiring additional treatment to achieve and maintain biochemical disease control, especially those with large tumors or persistent headaches (1, 52). Although the combination of pegvisomant and pasireotide appears to be the most effective treatment option for pharmacologically resistant acromegaly (53–55), such an approach is currently not reimbursed in Poland.

Biochemical response was achieved approximately six months after completion of radiotherapy. Therefore, the delayed therapeutic effect of radiotherapy may have contributed substantially to the subsequent normalization of IGF-1 levels. This suggests that the cumulative effects of the sequentially administered treatment modalities ultimately contributed to achieving biochemical disease control.

Given that a genetic cause is identified in nearly 50% of patients with pituitary gigantism, genetic assessment and testing are recommended for all pediatric patients presenting with GH excess (6). Pituitary gigantism may manifest either as isolated GH excess, most commonly associated with AIP mutations or X-linked acrogigantism, or as a component of inherited tumor predisposition syndromes, including McCune–Albright syndrome, Carney complex, multiple endocrine neoplasia type 1 (MEN1), and type 4 (MEN4), neurofibromatosis type 1 (NF1), *MAX*-associated disease, and paraganglioma/phaeochromocytoma and pituitary adenoma association (3Pas) (9, 22, 56).

Both the patient and her twin sister underwent conventional karyotyping and analysis using an NGS panel targeting genes associated with PitNET predisposition; however, no pathogenic variants were detected.

In conclusion this report illustrates the complexity of treating aggressive GH-secreting PitNETs in adolescent patient and reinforces the value of individualized, multidisciplinary management and prolonged follow-up. In cases refractory to surgery, radiotherapy, and standard medical therapy, combination treatment with pegvisomant and octreotide LAR may offer sustained biochemical disease control. As pituitary tumor enlargement may occur during pegvisomant monotherapy, regular MRI of the pituitary tumor is warranted.

## Data Availability

The original contributions presented in the study are included in the article/supplementary material. Further inquiries can be directed to the corresponding author.
